# Variants of the Coagulation and Inflammation Genes Are Replicably Associated with Myocardial Infarction and Epistatically Interact in Russians

**DOI:** 10.1371/journal.pone.0144190

**Published:** 2015-12-10

**Authors:** Rosa M. Barsova, Dmitrijs Lvovs, Boris V. Titov, Natalia A. Matveeva, Roman M. Shakhnovich, Tatiana S. Sukhinina, Nino G. Kukava, Mikhail Ya. Ruda, Irina M. Karamova, Timur R. Nasibullin, Olga E. Mustafina, German J. Osmak, Ekaterina Yu. Tsareva, Olga G. Kulakova, Alexander V. Favorov, Olga O. Favorova

**Affiliations:** 1 Institute of Experimental Cardiology, Russian Cardiology Scientific and Production Center, Moscow, Russia; 2 Department of Computational Biology, Vavilov Institute of General Genetics, Russian Academy of Sciences, Moscow, Russia; 3 Department of Molecular Biology and Medical Biotechnology, Pirogov Russian National Research Medical University, Moscow, Russia; 4 Department of Emergency Cardiology, Russian Cardiology Scientific and Production Center, Moscow, Russia; 5 Division of Cardiology, Republican Cardiology Health Center, Ufa, the Republic of Bashkortostan, Russia; 6 Department of Genomics, Institute of Biochemistry and Genetics, Ufa Research Center, Russian Academy of Sciences, Ufa, Russia; 7 Department of Oncology, Division of Biostatistics and Bioinformatics, Johns Hopkins School of Medicine, Baltimore, Maryland, United States of America; Shenzhen institutes of advanced technology, CHINA

## Abstract

**Background:**

In spite of progress in cardiovascular genetics, data on genetic background of myocardial infarction are still limited and contradictory. This applies as well to the genes involved in inflammation and coagulation processes, which play a crucial role in the disease etiopathogenesis.

**Methods and Results:**

In this study we found genetic variants of *TGFB1*, *FGB* and *CRP* genes associated with myocardial infarction in discovery and replication groups of Russian descent from the Moscow region and the Republic of Bashkortostan (325/185 and 220/197 samples, correspondingly). We also found and replicated biallelic combinations of *TGFB1* with *FGB*, *TGFB1* with *CRP* and *IFNG* with *PTGS1* genetic variants associated with myocardial infarction providing a detectable cumulative effect. We proposed an original two-component procedure for the analysis of nonlinear (epistatic) interactions between the genes in biallelic combinations and confirmed the epistasis hypothesis for the set of alleles of *IFNG* with *PTGS*. The procedure is applicable to any pair of logical variables, e.g. carriage of two sets of alleles. The composite model that included three single gene variants and the epistatic pair has AUC of 0.66 both in discovery and replication groups.

**Conclusions:**

The genetic impact of *TGFB1*, *FGB*, *CRP*, *IFNG*, and *PTGS* and/or their biallelic combinations on myocardial infarction was found and replicated in Russians. Evidence of epistatic interactions between *IFNG* with *PTGS* genes was obtained both in discovery and replication groups.

## Introduction

Myocardial infarction (MI) is the most severe type of coronary artery disease (CAD) and one of the leading causes of death worldwide. While CAD genetics is well studied at the genome-wide significance level [1], genetic data on MI are very limited. A few genome-wide association studies (GWASs) were performed for MI as a distinct phenotype [2], and only one MI-associated region, 9p21.3, was replicated in three GWASs [3–5] and validated in different countries including Russia [6]. Importantly, the association of genetic factors distinctly contributing to either development of coronary atherosclerosis or to MI with underlying coronary atherosclerosis was observed in [7].

Despite the progress in genetics of CAD, all genetic variants identified by GWASs explain together less than 20% of heritability, and a large portion of heritability remains missing for both CAD and MI. One likely reason for this is that, given the polygenic nature of complex traits and the relatively small observed effect sizes of the loci identified, many truly associated variants do not reach the stringent p-value threshold for genome-wide significance [8]. Another reason may be that the risk factors arise from cumulative effects of several loci on the phenotype as a result of nonlinear (epistatic) interactions between genes [9], which remain hidden from GWAS analysis [10].

In this study we made an effort to find genetic variants associated with MI using the “old-fashioned” candidate genes strategy. Atherosclerosis is driven by a chronic inflammatory process within an arterial wall initiated in response to damage of endothelial cells. Proinflammatory cytokines and chemokines, released from impaired endothelial cells, macrophages, or T cells, promote formation and growth of atherosclerotic plaque [11]. Proinflammatory factors may lead to rupture of the fibrous cap of atherosclerotic plaque and induce thrombosis, which is a dominant cause of acute coronary syndrome [12]. Thus, inflammation and coagulation play a dominant role in the pathogenesis of MI. Seventeen SNPs in/near 15 genes of coagulation and inflammation systems, which are known to influence the levels/activity of protein products involved in MI etiopathogenesis (S1 Table), were screened in patients with MI and population controls of Russian descent living in the Moscow region. Genetic variants found to be associated with MI in the discovery group were replicated in independent samples of MI patients and population controls from Bashkortostan region, men of the Russian descent only. Special attention was paid to identification of MI-associated composite markers, i.e. combinations of variants of different genes providing a detectable cumulative effect on the phenotype.

The cumulative effect of genetic variants may arise from summing up of their independent contributions or as a result of nonlinear (epistatic) interactions between the genes. Analysis of statistical interactions is nowadays a hot topic in bioinformatics research today (for example, see [13,14]). Nevertheless, we did not find any approved and unified procedure to detect epistasis in the way that could be easily shared between different studies.

In this work, we propose a novel procedure for testing the epistasis hypothesis in case-control studies. It is a combination of two previously described statistics that are based on different models: the synergy factor (SF) [15] and the exact three-way Fisher-like interaction numeric test [16] (FLINT). Both statistics were proposed for estimation of three-way interaction effects; they are analogous to two independent criteria–the odds ratio (OR) and Fisher’s exact test–commonly used in association studies of two-way interaction effects. The combination of these two criteria in an association study provides a measure for the effect size along with two different statistical characteristics of the effect reliability. This advantage of classical association study is inherited by the SF+FLINT procedure we propose here as a test for epistatic character of three-way interactions.

## Materials and Methods

### Subjects

The discovery MI group included the unrelated patients with acute MI under 70 years old and unrelated subjects as the control group; all individuals were residents of the Moscow region (Russian Federation). All 325 patients (236 men and 89 women, mean age ± standard deviation—53.2±9.9 years, in men– 50.6±9.6 years, in women—59.9±7.2 years) were clinically evaluated at the Russian Cardiology Scientific and Production Center, Moscow. The control group included 185 individuals (100 men and 85 women, mean age—60.0±13.3 years, 57.1±11.1 years in men, 63.3±11.3 years in women) subjected to medical examination to exclude MI and CAD.

The independent replication group included unrelated male patients under 65 years old with large MI and unrelated men of the control group; all individuals were residents of the Republic of Bashkortostan (Russian Federation). All 220 men with acute MI (mean age ± standard deviation 50.1±6.8 years) underwent a full clinical evaluation at the Republic Centre of Cardiology (Ufa, Russian Federation). The control group included 197 men under 67 years (45.3±7.9 years) without clinical evidence of cardiovascular disease.

The study subjects in both groups described themselves as ethnic Russians. Acute MI was diagnosed based on a rise in troponin I concentration or creatine kinase MB fraction activity accompanied by chest pain lasting longer than 30 minutes and emergence of new electrocardiogram abnormalities (pathological Q waves, ST elevation or depression). Clinical characteristics of the studied MI patients are shown in S2 Table. The study has been approved by the Ethics Committees of the Russian Cardiology Scientific and Production Center or the Institute of Biochemistry and Genetics USC RAS. Written informed consent was obtained from each participant in accordance with the Declaration of Helsinki.

### Genotyping

Genomic DNA was isolated from peripheral blood by phenol-chloroform extraction using standard procedures [17]. SNP genotyping was performed as described in the S3 Table. Quality for all assays was assessed by random selection of 20% or more samples for re-genotyping; in addition, sequence analysis of some amplicons was used to confirm the accuracy of the results obtained by PCR. No inconsistencies were observed.

### Statistical analysis

Deviations of the observed genotype frequencies from Hardy-Weinberg equilibrium and haplotype analysis were studied with Haploview 4.2 software.

The APSampler algorithm [18] was used to identify the alleles, genotypes and their combinations, whose carriage is associated with MI. The findings were then validated by standard statistical approaches: calculation of OR and 95% CI, the exact Fisher’s p-value (*p*_f_) and the permutation p-value (*p*_perm_) using the tools included in the APSampler software [18,19]. The permutation test evaluated the probability to obtain the same Fisher’s test result from randomized data of the same dimension as the input data. The differences were considered significant at CIs that did not cross 1, *p*_f_-values <0.01 and *p*_perm_-values <0.05. We considered a single SNP as associated with MI if association was significant either in recessive or dominant models.

To test the discovered allelic combinations for epistasis, we constructed 3-way contingency tables cross-tabulating all subjects from the study by joint carriership status for the two loci of interest and the outcome (case/control). The numeric detection of epistasis is performed by the two-statistic SF+FLINT procedure that consists of the evaluation of the Synergy Factor (SF) [15] and the exact Fisher-like interaction numeric test (FLINT) that is referred to as 3-way interaction test in sociology [16]. This procedure is realized as Flinte R package and Perl script [20]. If CI for SF did not cross 1, the pair of genetic variants was considered as interacting. SF>1 indicates a positive (synergistic) interaction, and SF<1 a negative (compensatory) interaction. FLINT was considered significant at exact interaction test *p*-value (p_FLINT_) <0.05. We considered interaction between two components of a biallelic MI-associated combination as significant if it was confirmed by both tests.

The performance of the predictive model was estimated by area under the curve (AUC) criteria for the receiver operating characteristic (ROC) curves of the logistic regression model that included the genetic markers as predictors and the MI occurrence as outcome. In order to evaluate gene-gender interaction we used logistic regression with interaction terms as well as the Flinte R package [20]. Logistic regression analyses were performed using R software (ver. 3.2.2) [21].

## Results

### Genetic associations with MI in discovery group from the Moscow region

#### Hardy–Weinberg equilibrium and linkage disequilibrium

All 17 SNPs were in Hardy–Weinberg equilibrium (*p*>0.01), except for *TGFB1* rs1800469 in MI patients (*p* = 0.0039) and for *IL10* rs1800896 in control group (*p =* 0.0075). *IL10* rs1800896 was excluded from the further analysis.

In LD analysis of the control group, we observed the linkage group of *FGA* rs6050 and *FGB* rs1800788 in fibrinogen gene cluster on chromosome 4 (D'<1, LOD>2) and of *LTA* rs909253 and *TNF* rs1800629 in *LTA*/*TNF* gene cluster on chromosome 6 (D'<1, LOD>2). We found no LD between rs1800469, rs1982073, and rs1800471 in *TGFB1* gene (D'<1, LOD<2) and between *PDE4D* rs152312 and *IL4* rs2243250 (chromosome 5) (S1 Fig).

#### Association with MI of variants of coagulation and inflammation genes

In discovery dataset, significant differences were found in carriage frequencies of alleles/genotypes of the following SNPs: *TGFB1* rs1982073, *FGB* rs1800788, and *CRP* rs1130864 (Table 1, left panel). The most significant positive association was observed for *TGFB1* rs1982073*TT (*p*_f_ *=* 0.00098, *p*_perm_ = 0.0082, OR = 1.84, 95%CI:1.26–2.68). Similarly, carriage of allele T (genotypes TT+CT) of *FGB* rs1800788 (*p*_f_ *=* 0.0012, *p*_perm_ = 0.0086, OR = 1.80, 95%CI:1.24–2.61) and of *CRP* rs1130864*TT (*p*_f_ *=* 0.0030, *p*_perm_ = 0.020, OR = 2.93, 95%CI:1.34–6.41) can also be considered risk factors for MI. When individuals were stratified according to gender, carriage of the aforementioned alleles/genotypes differed in MI patients and controls in both men and women with *p*_f_-values<0.05 and *p*_perm_ values>0.05 (not shown). Thus, there is no reason to assume gender specificity of the observed effects.

**Table 1:** SNPs positively associated with MI in discovery (325 MI patients and 185 controls from Moscow) and in independent replication group (220 MI patients and 197 controls from Bashkortostan, men only).

Gene, SNP	Carriage of risk genotypes (alleles)	Discovery group (Moscow)				Independent replication group (Bashkortostan, men only)			
		Frequency (case/control)	Fisher *p* value	Permutation *p* value*	OR (95% CI)	Frequency (case/control)	Fisher *p* value	Permutation *p* value*	OR (95% CI)
*TGFB1* rs1982073	TT	0.47/0.32	0.00098	0.0082	1.84 (1.26–2.68)	0.46/0.32	0.0025	0.013	1.79 (1.20–2.67)
*FGB* rs1800788	TT+CT (T)	0.52/0.38	0.0012	0.0086	1.80 (1.24–2.61)	0.51/0.37	0.0021	0.011	1.80 (1.21–2.66)
*CRP* rs1130864	TT	0.12/0.04	0.0030	0.020	2.93 (1.34–6.41)	0.15/0.04	0.00011	0.00026	4.17 (1.87–9.26)

* 100 permuted APSampler runs.

No significant differences were observed for the distribution of main conventional MI risk factors (smoking, age, essential hypertension, gender, diabetes) when stratifying carriers of TGFB1*TT, FGB*T or CRP*TT (S4 Table).

Other studied SNPs did not show any significant differences between MI patients and controls (S5 Table).

#### Allelic combinations associated with MI

The allelic combinations, which differ in MI patients and controls from the discovery dataset, according to both Fisher’s exact test and the permutation test, are shown in Table 2, left panel. The most significant association was shown for the carriage of protective biallelic combination *TGFB1* rs1982073*C with *CRP* rs1130864*C (*p*_perm_ = 0.00048, OR = 0.46, 95%CI:0.31–0.67); *p*_perm_-value for this combination was more significant than *p*_perm_ values for carriage of individual alleles TGFB1*C (*p*_perm_ = 0.0082) and CRP*C (*p*_perm_ = 0.020). The combination of *TGFB1* rs1982073*T and *FGB* rs1800788*T was found as a highly significant susceptibility pattern (*p*_perm_ = 0.00068, OR = 2.15, 95%CI:1.46–3.15). The *p*_perm_-value for this combination was less than for carriage of FGB*T individually (*p*_perm_ = 0.0086), whereas carriage of individual allele TGFB1*T was not significant according to both Fisher’s exact test and the permutation test. We also observed positive association of carriage of biallelic combination *IFNG* rs2430561*A and *PTGS1* rs3842787*T with MI (*p*_perm_ = 0.0095, OR = 2.97, 95%CI:1.41–6.23); however, the individual components of this allelic combination were not associated with MI. Thus, any genetic variant included in the biallelic set is characterized by the lower significance of association with MI than the set itself.

**Table 2:** Allelic combinations associated with MI according to APSampler analysis in discovery group (325 MI patients and 185 controls from Moscow) and in independent replication group (220 MI patients and 197 controls from Bashkortostan, men only).

Carriage of allele combinations	Discovery group (Moscow)					Independent replication group (Bashkortostan, men only)				
	Carriers, frequency (case/control)	*p*_f_ (*p*_perm_) values*	OR (95% CI)	SF (95% CI)	*p*_FLINT_ value**	Carriers, frequency (case/control)	*p*_f_ (*p*_perm_) values*	OR (95% CI)	SF (95% CI)	*p*_FLINT_ value**
*TGFB1* rs1982073*C + *CRP* rs1130864*C	0.49/0.66	0.000036 (0.00048)	0.46 (0.31–0.67)	0.39 (0.086–1.77)	0.26	0.45/0.65	0.000031 (0.000055)	0.44 (0.29–0.65)	0.66 (0.13–3.41)	0.67
*TGFB1* rs1982073*T + *FGB* rs1800788*T	0.48/0.30	0.000057 (0.00068)	2.15 (1.46–3.15)	2.22 (0.78–6.33)	0.18	0.45/0.30	0.00077 (0.004)	1.94 (1.30–2.92)	1.35 (0.44–4.15)	0.77
*IFNG* rs2430561*A + *PTGS1* rs3842787*T	0.14/0.05	0.0015 (0.0095)	2.97 (1.41–6.23)	5.18 (1.46–18.4)	0.018	0.16/0.07	0.0021 (0.011)	2.67 (1.37–5.22)	7.27 (1.72–30.8)	0.0012

* p_f_−Fisher *p* value; *p*_perm_−permutation *p* value (100 permuted APSampler runs).

** *p*_FLINT_−*p* value according to exact Fisher-like interaction numeric test (FLINT).

#### Detection of epistasis in allelic combinations

The epistasis hypothesis testing procedure that we propose here follows the paradigm of the common approach for testing the genotype-phenotype associations in case-control studies, which is based on two different statistical models. In this manner, two objectives are completed: first, the interaction hypotheses are tested using two different statistical models that in theory should make epistasis detection more robust, and second, two valuable and different, easily interpretable metrics of interaction between variables are provided. A set of Perl programs was created for automatic pairwise interaction hypothesis testing of genetic data, which is based on two statistics as described above, and these utilities are now available to download and use as part of the open-source APSampler project [19].

In cases of identified combinations (*TGFB1* rs1982073*C + *CRP* rs1130864*C) and (*TGFB1* rs1982073*T + *FGB* rs1800788*T), SFs were non-significant because their CIs cross 1; correspondingly, *p*_FLINT_-values were >0.05 (Table 2, left panel). These results are consistent with the lack of epistatic interactions between alleles in both combinations and possibly demonstrate their additive effects on MI susceptibility. At the same time, the components of MI risk combination (*IFNG* rs2430561*A + *PTGS1* rs3842787*T) demonstrated positive epistatic interaction according to both criteria: SF = 5.18 (95%CI:1.46–18.4), *p*_FLINT_ = 0.018.

### Replication of significant association results in the independent MI group from Bashkortostan

#### Analysis of association with MI of individual alleles and genotypes

SNPs *TGFB1* rs1982073, *FGB* rs1800788, *CRP* rs1130864, *IFNG* rs2430561 and *PTGS1* rs3842787 were genotyped in the independent groups of MI patients and population controls (men only). All SNPs were in Hardy–Weinberg equilibrium (*p*>0.01), except *CRP* rs1130864 (*p* = 0.0001) and *PTGS1* rs3842787 (*p* = 0.0086) in MI patients.

Genetic associations of *TGFB1* rs1982073, *FGB* rs1800788 and *CRP* rs1130864 with MI were independently replicated in this group (Table 1, right panel). Permutation *p*-value seems to be somewhat less reliable for MI-associated TGFB1*TT (*p*_perm_ = 0.013) and FGB*T (*p*_perm_ = 0.011) than in the discovery group; however the OR values are very close. Carriage of CRP*TT is the strongest MI risk factor in the replication group and is more significant than in the discovery group according to values of *p*_f_ (0.00011), *p*_perm_ (0.00026), and OR (4.17, 95%CI:1.87–9.26). Again, no statistical difference was observed in the distribution of main conventional risk factors by stratifying the carriers of risk genotypes (alleles) (S4 Table).

There were no differences in carriage of alleles/genotypes for *IFNG* rs2430561 and *PTGS1* rs3842787 between MI patients and control individuals (not shown).

#### Analysis of allelic combinations and epistatic interactions

The allelic combinations differing in MI patients and controls of the replication group are shown in Table 2, right panel; they are the same as in the discovery group. Biallelic combination (*TGFB1* rs1982073*C + *CRP* rs1130864*C) remained the most significant negatively associated pattern (*p*_perm_ = 0.000055, OR = 0.44, 95%CI:0.29–0.65). The values of *p*_perm_ and OR for positively associated biallelic combinations (*TGFB1* rs1982073*T + *FGB* rs1800788*T) and (*IFNG* rs2430561*A + *PTGS1* rs3842787*T) were comparable with those for the discovery group.

Exactly as in the discovery group, SFs and *p*_FLINT_-values for replicated combinations (TGFB1*C+CRP*C) and (TGFB1*T+FGB*T) were non-significant (Table 2, right panel). Therefore, we again did not observe epistatic interactions between alleles in these combinations, which possibly demonstrate their additive effects on MI susceptibility. Moreover, in the replication group we observed the same epistatically interacting pair of alleles (*IFNG* rs2430561*A + *PTGS1* rs3842787*T) as in the discovery group according to both criteria: SF = 7.27 (95%CI:1.72–30.8) and *p*_FLINT_ = 0.0012.

### Multiple logistic regression and ROC curve analysis of the identified genetic markers

Multiple logistic regression analysis was performed in the discovery group for the four identified genetic markers: the three single gene variants *TGFB1* rs1982073*TT, *FGB* rs1800788*T, *CRP* rs1130864*TT, and the biallelic combination *IFNG* rs2430561*A + *PTGS1* rs3842787*T. The last combination was recoded to form the fourth independent marker that took value 1 in case of the presence of both risk alleles, and 0 otherwise.

All the coefficients of the composite model (the one that included all four genetic markers) were significant at the *p*<0.01, with the largest effect sizes attributable to the *CRP* rs1130864*TT (regression coefficient β = 1.13, *p* = 0.005) and the combination *IFNG* rs2430561*A + *PTGS1* rs3842787*T (β = 1.19, *p* = 0.002) (S6 Table).

**Fig 1:**
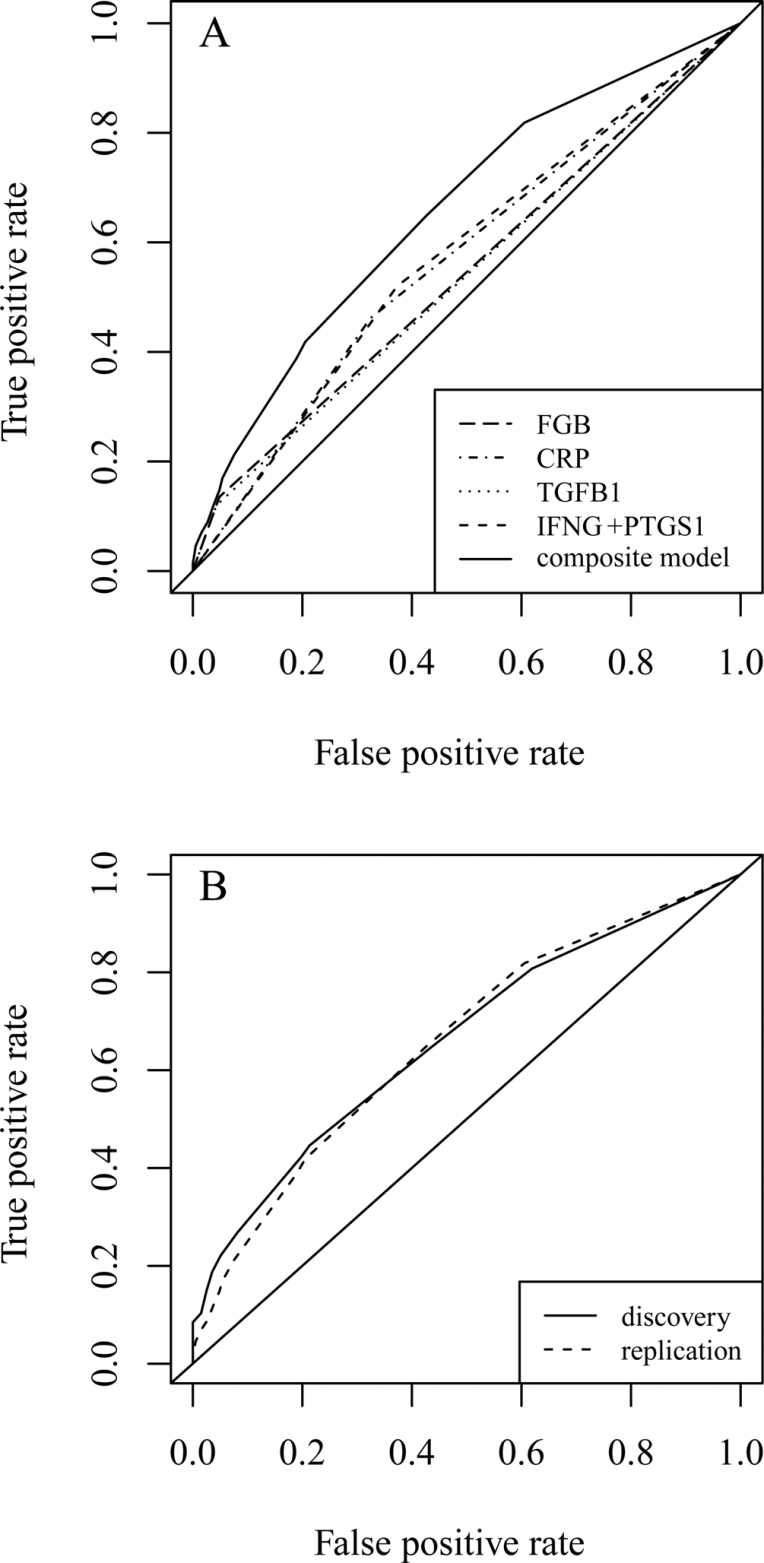
ROC curves demonstrate usefulness of the additive composite model built from all identified genetic markers. **A**. Comparing performance of the composite model to the performance of each single marker in the Moscow discovery sample. Combining the high specificity of *CRP* and *IFNG*+*PTGS* predictors (the left hump) with relatively high sensitivity of *TGFB1* and *FGB* (the right hump) yields a much better classifier. **B**. Performance of the model stays the same when tested on the independent replication sample (Bashkortostan).

To assess usefulness of the composite model to predict of individual risk of MI and to compare its performance to single genetic markers we have built the receiver operating characteristic (ROC) curves for each of the components of the model and for the resulting composite model (Fig 1A). The composite model has a rather moderate AUC of 0.66, although it does possess a predictive power superior to the single markers (S7 Table). The composite model performs very well when applied to the independent replication sample: the two ROC curves are almost identical (Fig 1B). The AUC of the model prediction in the replication group stays the same as in the discovery group (S7 Table).

## Discussion

In the discovery group from the Moscow region we performed the analysis of allele/genotype associations of 17 SNPs with the MI susceptibility. The SNPs were located in/near 15 genes; their effects on the level of gene transcription and/or mRNA stability and/or activity of protein product were described or assumed earlier (S1 Table). Both conventional (SNP-by-SNP) analysis and identification of MI-associated allelic combinations were conducted.

In the discovery group three SNPs were significantly associated with MI: *TGFB1* rs1982073, *FGB* rs1800788 and *CRP* rs1130864 (*p*_f_ <0.01 and *p*_perm_ <0.05). None of these SNPs was described as MI-associated in GWASs, performed in European [3,5] and Asian [4,22,23] populations. Nevertheless, all these loci were replicated in this study at the same significance level in an independent sample of the ethnical Russians, inhabitants of the Republic of Bashkortostan. This is compelling evidence of validity of these associations, at least for the Russian population. It should be mentioned that earlier we described MI-associated genetic variants *TGFB1* rs1982073 [24] and *CRP* rs1130864 [25] in the independent cohort of Moscow patients including individuals with the age at onset of more than 70 years (p_f_ ≤0.05).

TGF-β1 is a pleiotropic growth factor with dual (protective and detrimental) roles in atherogenesis, which are defined by the balance between Smad1/5- and Smad2/3-dependent signaling [26]. *TGFB1* polymorphism-related genotypes or haplotypes were described as associated with MI in men from Germany [27], four regions in France and Northern Ireland [28] and Japan [29], as well as in patients with early-onset MI from Italy (without gender stratification) [30]. Contradictory data on the positive or negative associations of the same SNP alleles with MI observed in these studies may be explained, at least in part, by the different genetic backgrounds of individuals under study influencing the balance of pro- and antiatherogenic effects of TGF-β1. *TGFB1* rs1982073 TT genotype was found to be the MI risk factor in Japanese [29] and in two independent samples of Russians in our study.

Fibrinogen participates in atherosclerotic plaque formation by modulation of endothelial function and promotion of smooth muscle cell proliferation and migration. Conversion of fibrinogen to fibrin plays an essential role in hemostasis and results in stabilization of the fibrin clot. Fibrinogen consists of three pairs of non-identical polypeptide chains, encoded by *FGA*, *FGB* and *FGG* genes, which form fibrinogen gene-cluster. Although plasma fibrinogen levels are related to cardiovascular risk, the data on the role of fibrinogen genetic variation in MI etiology remains inconsistent [31]. Some investigators have reported associations between SNPs or haplotypes of fibrinogen genes with MI [32,33], whereas the other studies did not replicate these associations [34–36]. In this study we observed the strong association of *FGB* rs1800788 but not of *FGA* rs6050 with MI in two geographically outlying groups despite a moderate linkage between these loci.

C-reactive protein (CRP) is a circulating biomarker of inflammation, and its level is a consistent risk factor for MI and cardiovascular disease [37]. rs1130864, which was studied in our work, is located at the position +1444 in the 3′ untranslated region of *CRP* gene, a regulatory region that plays a key role in controlling the gene expression. Earlier in Russian MI patients, we demonstrated the association of rs1130864 genotype TT both with higher level of CRP and MI risk [25]. Besides, genotype rs1130864*TT was recently found to be significantly associated with the risk of coronary heart disease risk in Northwest Indians [38] and with a larger coronary plaque volume in CAD patients [39].

The functional significance was described for all three MI-associated SNPs in this work (see S1 Table for references), and carriage of the risk alleles correlated with the plasma levels of the gene products expected to be involved in MI development. Namely, carriage of *TGFB1* rs1982073*TT correlates with the lower level of anti-inflammatory cytokine TGF-β1, carriage of *CRP* rs1130864*T is associated with increased C-reactive protein level, and carriage of *FGB* rs1800788*T correlates with the increased level of fibrinogen.

In addition to three MI-associated single SNPs, three highly significant MI-associated biallelic combinations–(*TGFB1* rs1982073*C + *CRP* rs1130864*C), (*TGFB1* rs1982073*T + *FGB* rs1800788*T) and (*IFNG* rs2430561*A + *PTGS1* rs3842787*T)–were found in the discovery group and replicated in the independent sample of Russians. To our knowledge, there is no generally accepted definition of allele/genotype combination (or gene set, or combining genetic profile) associated with the particular phenotype. Earlier we stated the concept of the minimal set (combination) of alleles as a genetic risk factor, which means that a combination is associated with the phenotype more reliably (e.g. with better *p*-value) than each of its components [9]. All biallelic combinations found in our study have lower Fisher’s and permutation *p*-values than their components (single alleles), both in discovery and replication groups (see data of Tables 1 and 2). The first two allelic sets include SNPs less reliably but significantly (at the level of *p*_f_-value <0.01) associated with MI individually. *IFNG* rs2430561 and *PTGS1* rs3842787 alleles, which are parts of the third set, were not significantly associated with MI by itself in both groups.

The obtained data on the association of distinct genetic variants and biallelic combinations with MI as well as data on their prognostic significance were very similar in the discovery and replication groups of Russians living in different regions of European Russia. At the same time, these groups of MI patients markedly differed from each other on several parameters (see S2 Table). Importantly, the discovery group from Moscow included men and women with the sex ratio of 2.5:1 as is typical for MI, whereas the replication group from Bashkortostan included men only. Since it was not possible to evaluate the found associations in women from Bashkortostan, we checked if the genetic effect is gender-moderated in the discovery group. To this end, we built a logistic regression model with gender, genetic factors and the gene-gender interaction terms as predictors and the disease status as the dependent variable and then fitted the model on the discovery group. The p-values for the nonzero interaction coefficient were >0.1 for all three MI-associated SNPs (S8 Table) that showed that the gender moderation did not exist. We checked the conclusion by the SF+FLINT procedure, with the same result.

The aim of the present study was also to evaluate and characterize possible gene-gene interactions between the components of allelic combinations, resulting in increased or decreased MI risk. For this purpose, we analyzed possible epistatic interactions between two components of detected combinations according to two criteria–SF and FLINT. The SF [15] is equivalent to the odds ratio (OR) in the two-way interaction analysis and is estimated together with its confidence interval (CI) using the model of independent parallel Poisson processes. Based on the logistic regression model, SF indicates the magnitude of the effect and does not change with varying sample size. SF is and has an important additional benefit of being applicable to the aggregated data and thus not requiring the initial data set. The SF statistic is more valid for case-control data [15] than its predecessor, the S statistic [40]. The second statistic, FLINT, which corresponds to the exact three-way interaction numeric test [16], is based on the model of uniform distribution of class populations with fixed marginal totals like the Fisher's exact test and also uses the aggregated data. The SF+FLINT criterion we proposed joins two statistics and requires both to be significant, so it is a more specific epistasis test than each of the methods applied separately.

Combinations (TGFB1*C+CRP*C) and (TGFB1*T+FGB*T) showed SF values, CI of which crossed the 1, and insignificant *p*_FLINT_ values in two independent groups. Therefore interactions in both combinations may be described as additive: in other words, the influences of alleles in these combinations on atherogenesis may simply add up. However, the sample size may be not enough to confirm the epistasis. However, the sample size may be not enough to confirm the epistasis.

In the case of combination (IFNG*A+PTGS1*T) the values of SF and FLINT *p-*values in the exact three-way interaction test, both in the discovery and replication groups, were consistent with the epistatic interaction between alleles in such a way that they form a new genetic composite marker of MI risk. Biallelic combination (IFNG*A + PTGS1*T), but not its components, was found as MI risk factor for the first time. However, for PTGS1*T, *p*_f_-values were less than 0.05 in both groups, whereas OR was equal to 1.79 (95%CI:1.01–3.15) in the discovery group, and to 1.71 (95%0.98–3.01) in the replication group. Previously, no association of *PTGS1* rs3842787 with MI was shown in White North Americans [41], whereas the results in Italian population indicated allele *IFNG* rs2430561*A as a genetic factor of MI risk [42].

The first component of combination (IFNG*A+PTGS1*T) codes pleiotropic cytokine interferon gamma, one of the major regulators of the functions and properties of all the cell types in the vessel wall, with both pro- and anti-atherogenic activities [43]. It was demonstrated that the transcription factor NFkappaB preferentially binds with allele *IFNG* rs2430561*T, which correlates with high interferon gamma expression level (S1 Table). The protein product of the second component of the biallelic combination–prostaglandin-endoperoxide synthase 1, also known as cyclooxygenase-1 –constitutively catalyzes the rate-limiting step of prostaglandin production and is a target for non-steroidal anti-inflammatory drugs [44]. The data about functional significance of SNP rs3842787 are limited and controversial. It was demonstrated that 50C>T change in *PTGS1* gene results in significantly increased COX-1 sensitivity to inhibition by indomethacin *in vitro* and by aspirin *ex vivo* (S1 Table).

**Fig 2:**
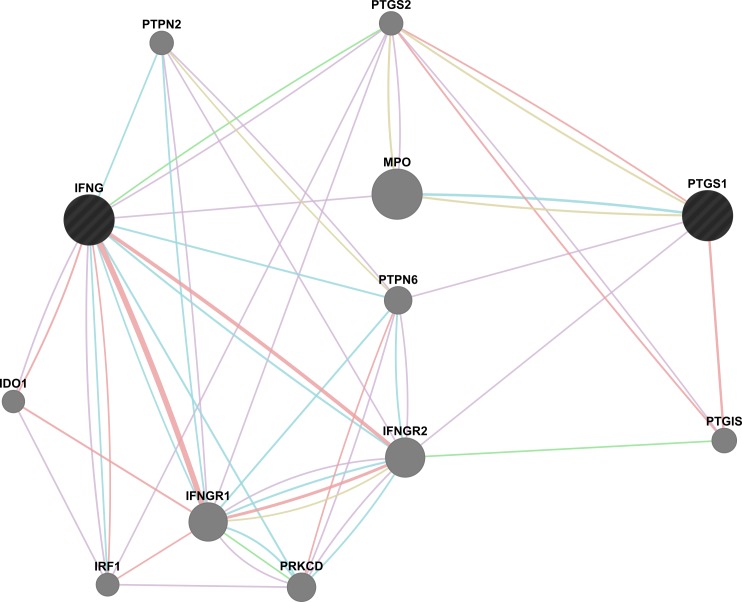
The map of possible interactions between components of MI-associated biallelic combination *IFNG* and *PTGS1* (black circles) and ten relative partners (gray circles) generated by GeneMania online software [45]. Possible physical interactions (pink), co-expression (violet), pathway (blue), genetic interactions (green), and shared protein domains (yellow) are shown. IDO1 –indoleamine 2,3–dioxygenase 1; IFNG–interferon gamma; IFNGR1 –interferon gamma receptor 1; IFNGR2 –interferon gamma receptor 2; IRF1 –interferon regulatory factor 1; MPO–myeloperoxidase; PTGIS–prostaglandin I2 (prostacyclin) synthase; PRKCD–protein kinase C delta; PTGS1 –prostaglandin–endoperoxide synthase 1; PTGS2 –prostaglandin–endoperoxide synthase 2; PTPN2 –protein tyrosine phosphatase, non–receptor type 2; PTPN6 –protein tyrosine phosphatase, non–receptor type 6.

To speculate about the mechanisms of detected epistatic interactions, we performed the network-based search of possible interactions between *IFNG* and *PTGS1* or their common pathways using GeneMania online software [45]. We did not observe any direct interactions between IFNG and PTGS1 (Fig 2). However, there is an indirect interplay of IFNG with PTGS1 via only one intermediate member–either PTGS2 (prostaglandin-endoperoxide synthase 2, or cyclooxygenase-2) or MPO (myeloperoxidase), or PTPN6 (protein tyrosine phosphatase, non-receptor type 6), or IFNGR2 (interferon gamma receptor 2). Basically, the network is clearly subdivided into two parts: prostaglandin synthesis pathway (cyclooxygenase pathway) and IFNG response pathway. It seems quite plausible that these interactions explain the existence of epistasis between *IFNG* and *PTGS1*; however further experimental evidence is required.

Several composite markers for MI and CAD using “a candidate-gene set” approach have been found. The choice of combining candidate genes was based on the previously obtained facts suggesting that the protein products of genes under study directly interact with a partner protein or its encoding gene. Therefore, if cumulative effects of gene polymorphisms are found, the reasons for gene-gene interactions are evident. It was found in the Rotterdam Study that the carriership of combination of CFH*402His and one of *CRP* haplotypes is associated with MI risk [46], and with the early atherogenic vascular changes (carotid artery compliance) in the men–participants of the Cardiovascular Risk in Young Finns Study [47]. Mannila et al. described epistatic effects of polymorphisms of fibrinogen genes, which contribute to MI risk. According to their data the *FGG* 9340T>C and *FGB* 1038G>A SNPs appeared to contribute to MI risk, explaining the association of *FGG-FGB* haplotypes with MI in the absence of effects of individual SNPs [33]. Gigante et al. showed that the interaction between *IL6* and *F2R* haplotypes increased the risk of MI in a study of 10 selected SNPs in Swedish men, in the absence of main effects [48]. At the same time, Lucas et al. analyzed gene-gene interactions in MI using GWAS data of SNPs that exhibit main effects with p-values <0.01; their massive study reports absence of replicable epistasis [49].

All in all, we revealed that the carriage of *TGFB1* rs1982073*TT, *FGB* rs1800788*T, *CRP* rs1130864*TT and epistatic allelic combination (*IFNG* rs2430561*A + *PTGS1* rs3842787*T) can be considered as independent risk factors for MI, at least in Russians. To check the possibility that our findings can be used for the creation of the MI prognostic test we performed the multiple logistic regression and ROC curve analysis of the identified genetic markers. We observed that the joint assessment of all the above risk factors can substantially increase the predictive power of the hypothetic MI risk test, as evidenced by a substantial improvement in AUC for the composite model as compared to the single predictors’ performance. It is important to note that the estimated predictive power of the risk factors found in this study was nearly the same in the discovery and replication samples; however, the resulting composite model is characterized by a rather moderate performance (AUC = 0.66).

Our results suggest also that independent genetic markers need not necessarily be separate genetic variants; those can also be synthetic variables composed from epistatically interacting individual markers that alone do not reach even the modest significance level. Another important source of potential improvement of the predictive power is the analysis of gene-environment interactions (G x E effects), which may increase the amount of gene variants associated with MI [50]. It is noteworthy that the two-component procedure proposed in this study is suitable not only for the analysis of interactions between the genes, but also of G x E interactions.

In the future, the discovered genetic variants replicably associated with MI can be used as substantial components for the creation and implementation of a prognostic test to determine individual risk of MI in Russians. However, these results require further investigation in populations of diverse descent, and prospective studies are of particular interest. In addition, the identification of new markers of genetic susceptibility to MI can expand the understanding of its pathogenesis and provide a basis for the search for new therapeutic targets.

## Supporting Information

S1 Fig Linkage analysis of studied SNPs located on chromosomes 4, 5, 6 and 19 (based on the evidence for control individuals of Russian descent from the Moscow region). White color indicates weak linkage (D'<1, LOD<2), pink color indicates moderate linkage (D'<1, LOD>2).
(TIF)

S1 Table Genetic polymorphisms selected for analysis of association with MI in individuals of Russian descent from the Moscow region. (DOC)

S2 Table Clinical profiles of the studied MI patients. (DOC)

S3 Table Genotyping methods applied for selected SNPs. (DOC)

S4 Table Carriage of risk genotypes in MI patients’ subgroups stratified by main conventional risk factors. (DOC)

S5 Table Case-control comparison of genotype frequencies of genetic variants, which were not associated with MI (Fisher *p*-values >0.01) in the discovery group from the Moscow region (325 MI patients and 185 controls). (DOC)

S6 Table The results of the fit on the discovery group of the composite regression model that includes all the four genetic markers as predictors. (DOC)

S7 Table The area under the curve (AUC) for the separate genetic risk factors and the composite model for the discovery group and the independent replication group. (DOC)

S8 Table *p*-Values for interaction of genetic markers with gender as tested in the discovery group. (DOC)
