# Supplementary figures and images for: Variants of the Coagulation and Inflammation Genes Are Replicably Associated with Myocardial Infarction and Epistatically Interact in Russians
Source: PLoS One. 2015 Dec 10;10(12):e0144190. doi: 10.1371/journal.pone.0144190 (PMC4675542; doi:10.1371/journal.pone.0144190)

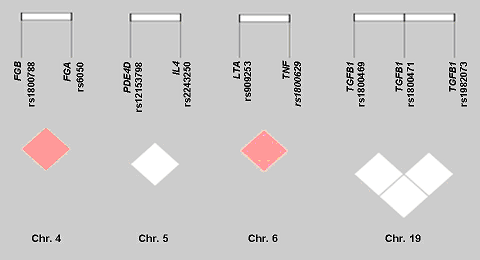

Supplement: S1 Fig — White color indicates weak linkage (D'<1, LOD<2), pink color indicates moderate linkage (D'<1, LOD>2). (TIF) [file pone.0144190.s001.TIF]
