# Supplementary material for: Variants of the Coagulation and Inflammation Genes Are Replicably Associated with Myocardial Infarction and Epistatically Interact in Russians
Source: PLoS One. 2015 Dec 10;10(12):e0144190. doi: 10.1371/journal.pone.0144190 (PMC4675542; doi:10.1371/journal.pone.0144190)
Supplement: S1 Table — (DOC) [file pone.0144190.s002.doc]

**Supporting Information**

**S1 Fig. Linkage analysis of studied SNPs located on chromosomes 4, 5, 6 and 19 (based on the evidence for control individuals of Russian descent from the Moscow region).** White color indicates weak linkage (D'<1, LOD<2), pink color indicates moderate linkage (D'<1, LOD>2).


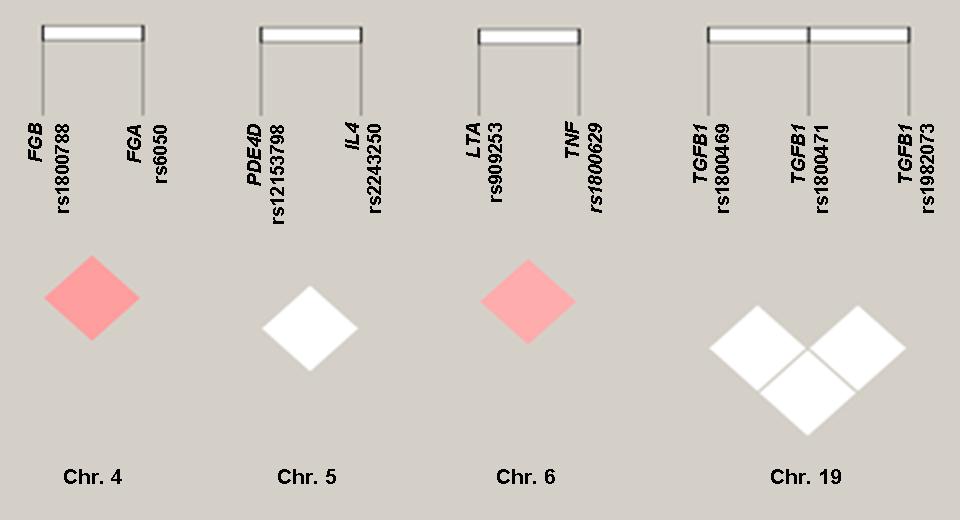


**S1 Table. Genetic polymorphisms selected for analysis of association with MI in individuals of Russian descent from the Moscow region**

| Gene symbol | Chr. location | Genetic variants* | SNP ID | Protein | Known (putative) effects on level/activity of the product |
| --- | --- | --- | --- | --- | --- |
| *CRP* | 1q21-q23 | 1444C>T | rs1130864 | C-reactive protein | Allele T is associated with increased plasma levels of C-reactive protein [1] |
| *IL10* | 1q32.1 | −1082G>A | rs1800896 | Interleukin 10 | Allele G determines the high-production of IL-10 [2] |
| *CTLA4* | 2q33.2 | 49A>G | rs231775 | Cytotoxic T-Lymphocyte Antigen 4 | CTLA-4 cell-surface expression is significantly increased in individuals carrying genotype A/A [3] |
| *CCR5* | 3p21.31 | Wild type→  32 base pair  deletion (w→del32) | rs333 | C-C chemokine receptor type 5 | ORF shift. The homozygous del32/del32 *CCR5* results in failure to express the receptor on the cell surface, while the heterozygous carriers express less receptor than wild-type homozygotes [4] |
| *FGA* | 4q31.3 | 4266A>G (Thr312Ala) | rs6050 | Fibrinogen alpha chain | Allele A correlates with stiffer clots [5] |
| *FGB* | 4q31.3 | −249C>T | rs1800788 | Fibrinogen beta chain | Allele T is associated with greater plasma fibrinogen levels [6] |
| *PDE4D* | 5q11.2-q12.1 | 41G>A | rs152312 | Phosphordiesterase 4D | Unknown |
| *IL4* | 5q31.1 | −590C>T | rs2243250 | Interleukin 4 | Allele T is associated with higher promoter activity and increased production of IL-4 as compared to C allele [7] |
| *TNF* | 6p21.3 | −308G>A | rs1800629 | Tumor necrosis factor (TNF), formerly known as TNF alpha | Allele A determines the enhanced production of TNF [8] |
| *LTA* | 6p21.3 | 252A>G | rs909253 | Lymphotoxin alpha | Allele G has been shown to correlate with elevated circulating lymphotoxin-alpha level [9] |
| *IL6* | 7p21 | −174G>C | rs1800795 | Interleukin 6 | Allele C was found to be associated with significantly lower levels of plasma IL-6 [10] |
| *PAI1* | 7q22.1 | −675 4G>5G | rs1799889 | Plasminogen activator inhibitor type 1 | Allele 4G is associated with greater circulating levels of PAI-1 [11] |
| *PTGS1* | 9q33.2 | 50C>T | rs3842787 | Prostaglandin-endoperoxide synthase 1, also known as Cyclooxygenase-1 (COX-1) | 50C>T change in *PTGS1* gene results in significantly increase COX-1 sensitivity to inhibition by indomethacin *in vitro* [12] and by aspirin *ex* vivo [13] |
| *IFNG* | 12q15 | 874A>T | rs2430561 | Interferon gamma | Allele T correlates with high IFN-gamma expression level [14] |
| *TGFB1* | 19q13.2 | −509C>T | rs1800469 | Transforming growth factor beta 1 | Allele T correlates with increased TGF-β1 plasma levels [15] |
|  |  | 869T>C (Leu10Pro) | rs1982073 |  | Allele C correlates with increased TGF-β1 plasma levels [16] |
|  |  | 915G>C (Arg25Pro) | rs1800471 |  | Genotype G/G correlates with increased TGF-β1 plasma levels [17] |

* All positions of the SNPs are indicated relative to transcriptional start sites except *CTLA4* SNP +49, where +49 is the position from translational start site.

ORF – open reading frame.

**References in S1 Table:**

1. Brull DJ, Serrano N, Zito F, Jones L, Montgomery HE, Rumley A, et al. Human CRP gene polymorphism influences CRP levels: implications for the prediction and pathogenesis of coronary heart disease. Arterioscler Thromb Vasc Biol. 2003;23: 2063–2069.
2. Lio D, Candore G, Crivello A, Scola L, Colonna-Romano G, Cavallone L, et al. Opposite effects of interleukin 10 common gene polymorphisms in cardiovascular diseases and in successful ageing: genetic background of male centenarians is protective against coronary heart disease. J Med Genet. 2004;41: 790–794.
3. Ligers A, Teleshova N, Masterman T, Huang WX, Hillert J. CTLA-4 gene expression is influenced by promoter and exon 1 polymorphisms. Genes Immun. 2001;2: 145–152.
4. Dean M, Carrington M, Winkler C, Huttley GA, Smith MW, Allikmets R, et al. Genetic restriction of HIV-1 infection and progression to AIDS by a deletion allele of the CKR5 structural gene. Hemophilia Growth and Development Study, Multicenter AIDS Cohort Study, Multicenter Hemophilia Cohort Study, San Francisco City Cohort, ALIVE Study. Science. 1996;273: 1856–1862.
5. Standeven KF, Grant PJ, Carter AM, Scheiner T, Weisel JW, Ariëns RA. Functional analysis of the fibrinogen alpha Thr312Ala polymorphism: effects on fibrin structure and function. Circulation. 2003; 107:2326–2330.
6. Liu Y, Berthier-Schaad Y, Fink NE, Fallin MD, Tracy RP, Klag MJ, et al. Beta–fibrinogen haplotypes and the risk for cardiovascular disease in a dialysis cohort. Am J Kidney Dis. 2005;46: 78–85.
7. Walley AJ, Cookson WO. Investigation of an interleukin-4 promoter polymorphism for association with asthma and atopy. J Med Genet. 1996;33: 689–692.
8. He B, Navikas V, Lundahl J, Söderström M, Hillert J. Tumor necrosis factor alpha-308 alleles in multiple sclerosis and optic neuritis. J Neuroimmunol. 1995;63: 143–147.
9. Cao C, Liu S, Lou SF, Liu T. The +252A/G polymorphism in the Lymphotoxin-α gene and the risk of non-Hodgkin lymphoma: a meta-analysis. Eur Rev Med Pharmacol Sci. 2014;18: 544–552.
10. Fishman D, Faulds G, Jeffery R, Mohamed-Ali V, Yudkin JS, Humphries S, et al. The effect of novel polymorphisms in the interleukin-6 (IL-6) gene on IL-6 transcription and plasma IL-6 levels, and an association with systemic-onset juvenile chronic arthritis. J Clin Invest. 1998;102: 1369–1376.
11. Nikolopoulos GK, Bagos PG, Tsangaris I, Tsiara CG, Kopterides P, Vaiopoulos A, et al. The association between plasminogen activator inhibitor type 1 (PAI-1) levels, PAI-1 4G/5G polymorphism, and myocardial infarction: a Mendelian randomization meta-analysis. Clin Chem Lab Med. 2014;52: 937–950. doi: 10.1515/cclm-2013-1124.
12. Lee CR, Bottone FG, Krahn JM, Li L, Mohrenweiser HW, Cook ME, et al. Identification and functional characterization of polymorphisms in human cyclooxygenase-1 (PTGS1). Pharmacogenet. Genomics. 2007;17: 145–160.
13. Halushka MK, Walker LP, Halushka PV. Genetic variation in cyclooxygenase 1: effects on response to aspirin. Clin Pharmacol Ther. 2003;73: 122–130.
14. Pravica V, Perrey C, Stevens A, Lee JH, Hutchinson IV. A single nucleotide polymorphism in the first intron of the human IFN-gamma gene: absolute correlation with a polymorphic CA microsatellite marker of high IFN-gamma production. Hum Immunol. 2000;61: 863–866.
15. Shah R, Hurley CK, Posch PE. A molecular mechanism for the differential regulation of TGF–beta–1 expression due to the common SNP –509C–T (c.–1347C–T). Hum. Genet. 2006;120: 461–469.
16. Yokota M, Ichihara S, Lin TL, Nakashima N, Yamada Y. Association of a T29→C polymorphism of the transforming growth factor–beta1 gene with genetic susceptibility to myocardial infarction in Japanese. Circulation. 2000;101: 2783–2787.
17. Awad MR, El-Gamel A, Hasleton P, Turner DM, Sinnott PJ, Hutchinson IV. Genotypic variation in the transforming growth factor-beta1 gene: association with transforming growth factor-beta1 production, fibrotic lung disease, and graft fibrosis after lung transplantation. Transplantation. 1998;66: 1014–1020.

** *p*_FLINT_ – *p* value according to exact Fisher-like interaction numeric test (FLINT).
