# Supplementary material for: Variants of the Coagulation and Inflammation Genes Are Replicably Associated with Myocardial Infarction and Epistatically Interact in Russians
Source: PLoS One. 2015 Dec 10;10(12):e0144190. doi: 10.1371/journal.pone.0144190 (PMC4675542; doi:10.1371/journal.pone.0144190)
Supplement: S2 Table — (DOC) [file pone.0144190.s003.doc]

**S2 Table.** **Clinical profiles of the studied MI patients**

|  | MI patients  (Moscow)  (n = 325) | MI patients  (Bashkortostan, men only)  (n = 220) |
| --- | --- | --- |
| Mean age of acute MI ± SD (years) | 53.2 ± 9.9 | 50.1 ± 6.8 |
| Smoking (%) | 68.2 | 86.3 |
| Essential hypertension before MI (%) | 68.0 | 57.3 |
| Diabetes mellitus before MI (%) | 23.4 | 0 |
| Total cholesterol ± SD (mg/dl) | 6.20 ± 1.45 | 4.93 ± 1.32 |
| LDL-cholesterol ± SD (mg/dl) | 3.88 ± 1.88 | 3.37 ± 1.22 |

** *p*_FLINT_ – *p* value according to exact Fisher-like interaction numeric test (FLINT).
