# Supplementary material for: Variants of the Coagulation and Inflammation Genes Are Replicably Associated with Myocardial Infarction and Epistatically Interact in Russians
Source: PLoS One. 2015 Dec 10;10(12):e0144190. doi: 10.1371/journal.pone.0144190 (PMC4675542; doi:10.1371/journal.pone.0144190)
Supplement: S3 Table — (DOC) [file pone.0144190.s004.doc]

**S3 Table. Genotyping methods applied for selected SNPs***

| Gene symbol | Genetic variants* | SNP ID | Genotyping method and primers |
| --- | --- | --- | --- |
| *CRP* | 1444C>T | rs1130864 | SSP-PCR  5’–TCGTTAACTATGCTGGGAAAC–3’ (SSP C),  5’–TCGTTAACTATGCTGGGAAAT–3’ (SSP T), 5’–TGATGAGCACTCTGGACCCAA–3’ (common primer), and  5’–CCCACGTCTCTGTCTCTGGT–3’ (common forward control primer) [1] |
| *IL10* | −1082G>A | rs1800896 | Real-time PCR |
| *CTLA4* | 49A>G | rs231775 | PCR-RFLP  5’-AAGGCTCAGCTGAACCTGGT,  5’-CTGCTGAAACAAATGAAACCC,  restricted by the *BstEII* restriction endonuclease (Sibenzyme, Russia) [2] |
| *CCR5* | Wild type→  32 base pair  deletion (w→del32) | rs333 | PCR  5’-AGGTCTTCATTACACCTGCAGC-3’ and  5’-CTTCTCATTTCGACACCGAAGC-3’ [2] |
| *FGA* | 4266A>G (Thr312Ala) | rs6050 | Real-time PCR |
| *FGB* | −249C>T | rs1800788 | Real-time PCR |
| *PDE4D* | 41G>A | rs152312 | Real-time PCR |
| *IL4* | −590C>T | rs2243250 | SSP-PCR  5’-CTAAACTTGGGAGAACATTGTC-3’ (SSP C),  5’-CTAAACTTGGGAGAACATTGTT 3’ (SSP T), and  5’-AGTACAGGTGGCATCTTGGAAA-3’ (common reverse primer) [2] |
| *TNF* | −308G>A | rs1800629 | Real-time PCR |
| *LTA* | 252A>G | rs909253 | PCR-RFLP  5'-GTGCTTCGTGCTTTGGAC TAC-3',  5'-GAGCTGGTGGGGACATGTCTG-3',  restricted by the *NcoI* restriction endonuclease (Sibenzyme, Russia) [3] |
| *IL6* | −174G>C | rs1800795 | Real-time PCR |
| *PAI1* | −6754G>5G | rs1799889 | Real-time PCR |
| *PTGS1* | 50C>T | rs3842787 | Real-time PCR |
| *IFNG* | 874A>T | rs2430561 | SSP-PCR  5’-TTCTTACAACACAAAATCAAATCT -3’ (SSP T),  5’-TTCTTACAACACAAAATCAAATCA -3’ (SSP A),  5’-TCAACAAAGCTGATACTCCA-3’ (common primer)  and 5’-GTTGCTCACTGGGATTTTTGG-3’ (common forward control primer) [4] |
| *TGFB1* | −509C>T | rs1800469 | SSP-PCR  5’-GGGCAACAGGACACCTGAA-3’(SSP T),  5’-GGGCAACAGGACACCTGAG-3’ (SSP C), and  5’-AAGGCATGGCACCGCTTCTG-3’ (common primer) [2] |
|  | 869T>C (Leu10Pro) | rs1982073 | SSP-PCR  5’-AGCAGCGGTAGCAGCAGCA-3’ (SSP T),  5’-GCAGCGGTAGCAGCAGCG-3’ (SSP C) and  5’-CT ACCTTTT GCC GGGAGACC -3’ (common primer) [5] |
|  | 915G>C (Arg25Pro) | rs1800471 | SSP-PCR  5’- TGGTGCTGACGCCTGGCCG-3’ (SSP G), 5’-TGGTGCTGACGCCTGGCCC-3’ (SSP C), and 5’-GGCGAGCCGCAGCTTGGACA-3’ (common primer) [5] |

SSP-PCR – Single Specific Primer-Polymerase Chain Reaction.

Real-time PCR – real-time polymerase chain reaction using TaqMan® SNP Genotyping Assay (Applied Biosystems, USA) according to the instruction of manufacturer.

PCR-RFLP – Restriction Fragment Length Polymorphism Analysis of PCR-Amplified Fragments.

* In all cases, except real-time PCR analysis, the presence of PCR products was checked by 2–3% agarose gel electrophoresis with and visualized under UV light after staining with ethidium bromide.

**References in S3 Table:**

1. Shakhnovich RM, Sukhinina TS, Barsova RM, Sudomoina MA, Rybalkin IN, Shreider EV, et al. [Polymorphism C1444T of C-reactive protein gene and C-reactive protein concentration in blood serum of healthy people and patients with myocardial infarction]. Kardiologiia. 2010;50: 4–12. Russian
2. Makarycheva OY, Tsareva EY, Sudomoina MA Kulakova OG,Titov BV, Bykova OV et al. Family Analysis of Linkage and Association of HLA-DRB1, CTLA4, TGFB1, IL4, CCR5, RANTES, MMP9 and TIMP1 Gene Polymorphisms with Multiple Sclerosis. Acta Naturae. 2011;3: 85–92.
3. Sudomoina MA, Sukhinina TS, Barsova RM, Favorov AV, Shakhnovich RM, Titov BV, et al. [Complex analysis of association of inflammation genes with myocardial infarction]. Mol Biol (Mosk). 2010;44: 463–471. Russian
4. Tsareva EIu, Kulakova OG, Makarycheva OIu, Boĭko AN, Shchur SG, Lashch NIu, et al. [Pharmacogenomics of multiple sclerosis: association of immune response genes polymorphism with copaxone treatment efficacy]. Mol Biol (Mosk). 2011;45: 963–972. Russian
5. Barsova RM, Titov BV, Matveeva NA, Favorov AV, Sukhinina TS, Shahnovich RM, et al. Contribution of the TGFB1 Gene  to Myocardial Infarction Susceptibility. Acta Naturae. 2012;4: 74–79.
