# Supplementary material for: Variants of the Coagulation and Inflammation Genes Are Replicably Associated with Myocardial Infarction and Epistatically Interact in Russians
Source: PLoS One. 2015 Dec 10;10(12):e0144190. doi: 10.1371/journal.pone.0144190 (PMC4675542; doi:10.1371/journal.pone.0144190)
Supplement: S4 Table — (DOC) [file pone.0144190.s005.doc]

**S4 Table. Carriage of risk genotypes in MI patients’ subgroups stratified by main conventional risk factors**

| Carriage of risk genotypes (alleles) | MI patients (Moscow) | | | | | | | | | | MI patients (Bashkortostan, men only) * | | | | | |
| --- | --- | --- | --- | --- | --- | --- | --- | --- | --- | --- | --- | --- | --- | --- | --- | --- |
|  | Smoking | | Age, years | | Essential hypertension | | Gender | | Diabetes mellitus | | Smoking | | Age, years | | Essential hypertension | |
|  | Carriers, frequency  (Yes/No) | Fisher  *p* value | Carriers, frequency  (≤55/>55) | Fisher  *p* value | Carriers, frequency  (Yes/No) | Fisher  p value | Carriers, frequency  (≤55/>55) | Fisher  *p* value | Carriers, frequency  (Yes/No) | Fisher  *p* value | Carriers, frequency (Yes/No) | Fisher  *p* value | Carriers, frequency  (≤55/>55) | Fisher  *p* value | Carriers, frequency  (Yes/No) | Fisher  *p* value |
| *TGFB1*  rs1982073***TT | 0.49/0.43 | 0.31 | 0.48/0.45 | 0.65 | 0.43/0.55 | 0.07 | 0.45/0.53 | 0.33 | 0.48/0.47 | 1.00 | 0.48/0.36 | 0.31 | 0.45/0.53 | 0.33 | 0.18/0.11 | 0.18 |
| *FGB*  rs1800788***T | 0.57/0.51 | 0.31 | 0.55/0.49 | 0.26 | 0.50/0.58 | 0.22 | 0.51/0.53 | 0.87 | 0.53/0.53 | 1.00 | 0.51/0.50 | 1.00 | 0.51/0.53 | 0.87 | 0.50/0.52 | 0.78 |
| *CRP*  rs1130864***TT | 0.13/0.11 | 0.85 | 0.48/0.45 | 0.65 | 0.11/0.15 | 0.35 | 0.16/0.13 | 0.82 | 0.08/0.13 | 0.44 | 0.16/0.11 | 0.78 | 0.16/0.13 | 0.82 | 0.45/0.48 | 0.78 |

Two-tailed Fisher *p*-values are presented

* Only patients without diabetes mellitus were included
