# Supplementary material for: Variants of the Coagulation and Inflammation Genes Are Replicably Associated with Myocardial Infarction and Epistatically Interact in Russians
Source: PLoS One. 2015 Dec 10;10(12):e0144190. doi: 10.1371/journal.pone.0144190 (PMC4675542; doi:10.1371/journal.pone.0144190)
Supplement: S5 Table — (DOC) [file pone.0144190.s006.doc]

**S5 Table. Case-control comparison of genotype frequencies of genetic variants, which were not associated with MI (Fisher p-values >0.01) in the discovery group from the Moscow region (325 MI patients and 185 controls)**

| Gene  SNP | Carriage of genotypes (alleles) | Frequency  in MI cases | Frequency  in controls |
| --- | --- | --- | --- |
| *IL10*  rs1800896 | GG | 0.19 | 0.15 |
|  | GA | 0.50 | 0.60 |
|  | AA | 0.31 | 0.25 |
|  | GG +GA (G) | 0.69 | 0.75 |
|  | GA+ AA (A) | 0.81 | 0.85 |
| *CTLA4*  rs231775 | AA | 0.27 | 0.30 |
|  | AG | 0.49 | 0.51 |
|  | GG | 0.24 | 0.19 |
|  | AA + AG (A) | 0.76 | 0.81 |
|  | AG + GG (G) | 0.73 | 0.70 |
| *CCR5*delta32  rs333 | ww | 0.82 | 0.79 |
|  | wd | 0.17 | 0.20 |
|  | dd | 0.01 | 0.01 |
|  | ww + wd (w) | 0.99 | 0.99 |
|  | wd + dd (d) | 0.18 | 0.21 |
| *FGA*  rs6050 | AA | 0.52 | 0.56 |
|  | AG | 0.41 | 0.37 |
|  | GG | 0.07 | 0.07 |
|  | AA + AG (A) | 0.93 | 0.93 |
|  | AG + GG (G) | 0.48 | 0.44 |
| *PDE4D*  rs152312 | GG | 0.81 | 0.81 |
|  | GA | 0.17 | 0.18 |
|  | AA | 0.02 | 0.01 |
|  | GG + GA (G) | 0.98 | 0.99 |
|  | GA + AA (A) | 0.19 | 0.19 |
| *IL4*  rs2243250 | CC | 0.66 | 0.63 |
|  | CT | 0.30 | 0.34 |
|  | TT | 0.04 | 0.03 |
|  | CC + CT (C) | 0.96 | 0.97 |
|  | CT + TT (T) | 0.34 | 0.37 |
| *TNF*  rs1800629 | GG | 0.76 | 0.82 |
|  | GA | 0.23 | 0.18 |
|  | AA | 0.01 | 0.00 |
|  | GG +GA (G) | 0.99 | 1.00 |
|  | GA+ AA (A) | 0.24 | 0.18 |
| *LTA*  rs909253 | AA | 0.52 | 0.57 |
|  | AG | 0.43 | 0.39 |
|  | GG | 0.06 | 0.04 |
|  | AA + AG (A) | 0.95 | 0.96 |
|  | AG + GG (G) | 0.49 | 0.43 |
| *IL6*  rs1800795 | GG | 0.31 | 0.33 |
|  | GC | 0.47 | 0.48 |
|  | CC | 0.22 | 0.19 |
|  | GG + GC (G) | 0.78 | 0.81 |
|  | GC + CC (C) | 0.69 | 0.67 |
| *PAI1*  rs1799889 | 4G4G | 0.32 | 0.33 |
|  | 4G5G | 0.45 | 0.46 |
|  | 5G5G | 0.23 | 0.21 |
|  | 4G4G + 4G5G (4G) | 0.77 | 0.79 |
|  | 4G5G + 5G5G (5G) | 0.68 | 0.67 |
| *PTGS1*  rs3842787 | CC | 0.83 | 0.90 |
|  | CT | 0.17 | 0.10 |
|  | TT | 0.00 | 0.00 |
|  | CC + CT (C) | 1.00 | 1.00 |
|  | CT + TT (T) | 0.17 | 0.10 |
| *IFNG*  rs2430561 | AA | 0.34 | 0.34 |
|  | AT | 0.48 | 0.47 |
|  | TT | 0.18 | 0.19 |
|  | AA + AT (A) | 0.82 | 0.81 |
|  | AT + TT (T) | 0.66 | 0.66 |
| *TGFB1*  −509C>T  rs1800469 | CC | 0.32 | 0.40 |
|  | CT | 0.56 | 0.50 |
|  | TT | 0.12 | 0.10 |
|  | CC + CT (C) | 0.88 | 0.90 |
|  | TT + CT (T) | 0.68 | 0.60 |
| *TGFB1*  915G>C  rs1800471 | CC | 0.01 | 0.01 |
|  | CG | 0.09 | 0.12 |
|  | GG | 0.90 | 0.87 |
|  | CC + CG (C) | 0.10 | 0.13 |
|  | GG+ CG (G) | 0.99 | 0.99 |
