# Supplementary material for: Variants of the Coagulation and Inflammation Genes Are Replicably Associated with Myocardial Infarction and Epistatically Interact in Russians
Source: PLoS One. 2015 Dec 10;10(12):e0144190. doi: 10.1371/journal.pone.0144190 (PMC4675542; doi:10.1371/journal.pone.0144190)
Supplement: S6 Table — (DOC) [file pone.0144190.s007.doc]

**S6 Table. The results of the fit on the discovery group of the composite regression model that includes all the four genetic markers as predictors**

| Predictor | Regression coefficient β | *p*-value |
| --- | --- | --- |
| *TGFB1* rs1982073*TT | 0.56 | 0.005 |
| *FGB* rs1800788*T | 0.63 | 0.001 |
| *CRP* rs1130864*TT | 1.13 | 0.005 |
| *IFNG* rs2430561*A + *PTGS1* rs3842787*T | 1.19 | 0.002 |
| Intercept | -0.123 | 0.4 |
