# Supplementary material for: Variants of the Coagulation and Inflammation Genes Are Replicably Associated with Myocardial Infarction and Epistatically Interact in Russians
Source: PLoS One. 2015 Dec 10;10(12):e0144190. doi: 10.1371/journal.pone.0144190 (PMC4675542; doi:10.1371/journal.pone.0144190)
Supplement: S7 Table — (DOC) [file pone.0144190.s008.doc]

**S7 Table. The area under the curve (AUC) for the separate genetic risk factors and the composite model for the discovery group and the independent replication group**

| Genetic risk factor | Discovery group (Moscow) | Replication group (Bashkortostan, men only) |
| --- | --- | --- |
|  | AUC | |
| *TGFB1* rs1982073*TT | 0.57 | 0.57 |
| *FGB* rs1800788*(T) | 0.57 | 0.57 |
| *CRP* rs1130864*TT | 0.54 | 0.55 |
| *IFNG* rs2430561*A + *PTGS1* rs3842787*T | 0.54 | 0.55 |
| Composite model | 0.66 | 0.66 |
