# Supplementary material for: Variants of the Coagulation and Inflammation Genes Are Replicably Associated with Myocardial Infarction and Epistatically Interact in Russians
Source: PLoS One. 2015 Dec 10;10(12):e0144190. doi: 10.1371/journal.pone.0144190 (PMC4675542; doi:10.1371/journal.pone.0144190)
Supplement: S8 Table — (DOC) [file pone.0144190.s009.doc]

**S8 Table. *p***-**values for interaction of genetic markers with gender as tested in the discovery group**

| Genes | *p*_r_ value* | *p*_FLINT_ value** | SF (95% CI) |
| --- | --- | --- | --- |
| *CRP* | 0.80 | 1 | 1.25 (0.23 – 6.85) |
| *TGFB1* | 0.66 | 0.69 | 1.2 (0.54 – 2.7) |
| *FGB* | 0.15 | 0.17 | 0.56 (0.26 – 1.22) |
| *IFNG+PTGS* | 0.50 | 0.70 | 1.69 (0.37 – 7.64) |

** p*_r_ –*p* value for interaction term of the logistic regression model.

** *p*_FLINT_ – *p* value according to exact Fisher-like interaction numeric test (FLINT).
